# PRI-724 and IWP-O1 Wnt Signaling Pathway Inhibitors Modulate the Expression of Glycolytic Enzymes in Tongue Cancer Cell Lines

**DOI:** 10.3390/cimb45120599

**Published:** 2023-11-29

**Authors:** Robert Kleszcz, Jarosław Paluszczak, Marta Belka, Violetta Krajka-Kuźniak

**Affiliations:** Chair and Department of Pharmaceutical Biochemistry, Poznan University of Medical Sciences, Rokietnicka 3, 60-806 Poznań, Poland; paluszcz@ump.edu.pl (J.P.); mbelka@ump.edu.pl (M.B.); vkrajka@ump.edu.pl (V.K.-K.)

**Keywords:** Wnt signaling, the Warburg effect, aerobic glycolysis, head and neck cancer, tongue cancer, PRI-724, IWP-O1, β-catenin, pyruvate kinase, lactate dehydrogenase

## Abstract

The dysregulation of energetic metabolism is one of the hallmarks of cancer cells. Indeed, the growth of head and neck squamous cell carcinoma (HNSCC) cells depends heavily on glycolytic activity, which can be considered a potential therapeutic target. Wnt signaling is one of the pathways that undergoes upregulation in HNSCC. Our previous studies have shown that Wnt signaling inhibitors—PRI-724 and IWP-O1—attenuate tongue SCC survival and reduce glucose uptake and lactate release. The aim of this research was to further evaluate the possible mechanisms of the previously observed effects. We assessed the effect of PRI-724 and IWP-O1 on the expression of selected glycolytic enzymes: phosphofructokinase M, pyruvate kinase M2, and lactate dehydrogenase. Relative transcript expression was assessed by real-time PCR, and protein levels by Western blot. Moreover, clinical data concerning mRNA and protein expression, gene promoter methylation, and HNSCC patients’ survival time were analyzed by the UALCAN tool, and protein–protein interaction was assessed using the STRING database. Experimental and bioinformatic data confirmed the relation between Wnt signaling and glycolytic enzymes in tongue cancer cells and HNSCC clinical samples. Overall, the inhibition of glucose metabolism by Wnt signaling inhibitors is a promising mode of action against tongue cancer cells.

## 1. Introduction

The development of cancer is a complex, multistep process. Tumor cells gain and present characteristic hallmarks. The foundations of cancer cells’ characteristics include their self-sufficiency in growth signals with concomitant insensitivity to antigrowth signals, evasion of apoptosis with limitless proliferation, promotion of angiogenesis, local invasion, and systemic metastasis [1]. The first twenty years of the 21st century allowed significant progress in cancer biology. Currently, cancer cells are also described by genome instability and mutations co-existing with non-mutational epigenetic reprogramming, inflammatory microenvironments supporting the avoidance of the immune response, unlocked phenotypic plasticity, senescence of cells, abnormalities in residing microbiomes and neuronal signaling, and finally, dysregulated cellular metabolism [2,3].

Changes in energy metabolism in cancer cells are known as the Warburg effect or aerobic glycolysis. Over a hundred years since its discovery by Otto Warburg, the understanding of this hallmark of cancer has been improved and revised many times. The enhanced activity of the glycolytic pathway enables effective redirection of metabolites, including standard oxidative fates of pyruvate via entering the citric acid cycle, the production of lactate (and related extracellular acidification), or the use of glycolytic intermediates for the biosynthesis of molecules essential for cancer cell growth and division, e.g., via the pentose phosphate pathway. Beyond energy (ATP) synthesis, the reorganization of energy metabolism is engaged in the modulation of cancer cell signaling, e.g., by promoting the formation of reactive oxygen species (ROS), modifying epigenetic mechanisms of gene expression, and promoting the expression and activity of metabolism-related transcription factors, like c-Myc and Hif-1α [4,5]. Currently, experimental observations indicate that the Warburg effect is a consequence of cancer phenotype development, and an adaptation to the hypoxic microenvironment by tumor cells is crucial during cancer progression [5].

Head and neck squamous cell carcinoma (HNSCC) is one of the leading tumor types worldwide [6]. Its occurrence depends on both genetic and epigenetic abnormalities, and in a subset of cases, it is caused by human papillomavirus (HPV) infection. Importantly, HPV-negative HNSCC tumors present a worse prognosis and higher diversity in molecular changes, for instance, in the activity of pro-tumorigenic signaling pathways [7]. The significance of particular hallmarks of cancer among HNSCC differs, and not all are experimentally well-proven [8]. Regarding metabolic alterations, accelerated glycolysis was vital for HNSCC tumor progression and the avoidance of immune system responses [9]. Moreover, high glycolytic activity measured based on 18F-FDG positron-emission tomography was a negative prognostic factor for HNSCC patients [10]. Thus, metabolic pathways are now considered potential targets of HNSCC therapy [11,12].

Several molecular pathways were described to be connected with HNSCC development. Our previous research focusing on Wnt signaling and the Akt kinase revealed that Wnt pathway inhibitors—PRI-724 and IWP-O1—had minor to moderate influence on tongue cancer cell proliferation, cytotoxicity, and apoptosis, while Akt inhibitors led to stronger effects on cell survival. The combinations of Wnt signaling and Akt inhibitors partly improved the anticancer effects [13]. Akt kinase and Wnt pathways were shown to be engaged in controlling cellular energetics [14,15]. Still, their role in modulating glycolytic activity in HNSCC has not been comprehensively described. Previously, we showed that PRI-724 and IWP-O1 significantly reduced glucose uptake, and PRI-724 also reduced lactate release in tongue cancer cells. In contrast, Akt kinase inhibition did not show any substantial effects [13]. Similar advantageous effects were observed in experiments using combinations of Wnt signaling inhibitors (PRI-724 and IWP-O1) with direct glycolytic inhibitors (2-deoxyglucose and lonidamine), since individually applied glycolytic inhibitors had limited influence on glucose consumption and lactate release [16].

Based on our previous findings, this study aimed to gain more insight into the mechanism of the observed functional effects of Wnt inhibitors on glucose metabolism. For this purpose, we experimentally assessed the influence of PRI-724, IWP-O1, and Akt inhibitor on the expression of key glycolytic enzymes in CAL 27, SCC-25, and BICR 22 tongue squamous cell carcinoma cell lines. Additionally, bioinformatic analyses were performed in order to further validate the inter-relationship between the aberrations in Wnt signaling and glycolysis, and its significance for disease progression. This allowed the clinical context to be brought into the in vitro experimental results.

## 2. Materials and Methods

### 2.1. Cells and Culture Conditions

Commercially available tongue squamous cell carcinoma cell lines were used in the experiments: CAL 27 and SCC-25 cell lines (derived from primary tumors) were purchased from the American Type Culture Collection (ATCC, Manassas, VA, USA), while BICR 22 cells (derived from a tongue cancer lymph node metastasis) were purchased from the European Collection of Authenticated Cell Cultures (ECACC, Porton Down, Wiltshire, UK).

The CAL 27 and BICR 22 cells were grown in high-glucose DMEM medium (Biowest, Nuaillé, France), supplemented with 10% FBS (EURx, Gdańsk, Poland) and a 1% antibiotic solution (penicillin and streptomycin; Biowest, Nuaillé, France). SCC-25 cells were grown in a 1:1 mixture of DMEM medium with a F12 medium containing 1.2 g/L sodium bicarbonate, 2.5 mM L-glutamine, 15 mM HEPES, and 0.5 mM sodium pyruvate (Biowest, Nuaillé, France) supplemented with 10% FBS (EURx, Gdańsk, Poland), a 1% antibiotic solution (penicillin and streptomycin; Biowest, Nuaillé, France), and 400 ng/mL hydrocortisone (Sigma-Aldrich, St. Louis, MO, USA). All cells were cultured under standard conditions (37 °C, 5% CO_2_, 95% humidity) in an incubator (Memmert, Schwabach, Germany).

### 2.2. Inhibitors

Three small-molecule inhibitors were used in the experiments. PRI-724 (Selleck Chemicals, Pittsburgh, PA, USA) and IWP-O1 (Sigma-Aldrich, St. Louis, MO, USA) are Wnt signaling inhibitors, which target the interaction between β-catenin and CREB binding protein (CREBBP) or the activity of Porcupine (*O*-acyltransferase), respectively. Moreover, Akt inhibitor X (Sigma-Aldrich, St. Louis, MO, USA) was used to inhibit the Akt kinase. Stock solutions of the compounds were prepared in DMSO and stored in aliquots at −20 °C.

In all the experiments, IC25 concentrations of individual compounds were used, and combinations were composed of two compounds with equal potency (IC25 + IC25). The IC25 values were previously determined by the MTS viability assay [13], and amount to: PRI-724 = 2.6 µM (CAL 27 cells), 0.85 µM (SCC-25), and 3.0 µM (BICR 22 cells); IWP-O1 = 1.0 µM (CAL 27 cells), 10.0 µM (SCC-25 cells), and 3.0 µM (BICR 22 cells); Akt inhibitor = 5.0 µM (CAL 27 cells), 7.0 µM (SCC-25 cells), and 4.0 µM (BICR 22 cells).

### 2.3. Isolation of RNA, Reverse Transcription, and Quantitative Real-Time PCR

Total RNA was isolated from cells treated with the compounds for 48 h using the Universal RNA Purification Kit (EURx, Gdańsk, Poland), and samples were subjected to reverse transcription using the RevertAid First Strand cDNA Synthesis Kit (Thermo Scientific, Waltham, MA, USA), in accordance with the manufacturer’s instructions.

For the qR-T PCR analyses, the SG qPCR Master Mix (EURx, Gdańsk, Poland) and LightCycler 96 (Roche, Basel, Switzerland) were used. The initial enzyme activation at 95 °C lasted 10 min, and was followed by 40 three-step cycles consisting of denaturation (95 °C for 15 s), primer annealing (56 °C for 30 s), elongation (72 °C for 30 s with fluorescence measurement), and subsequent melting curve analysis. The relative mRNA expression of phosphofructokinase M—*PFKM*, pyruvate kinase M2—*PKM2*, and lactate dehydrogenase A—*LDHA* was determined. The TATA-box-binding protein (*TBP*) expression was used to normalize the data, and the ΔΔCt method served for fold-change quantification. Three independent experiments were performed with three technical repeats for each sample during qR-T PCR. The sequences of the primers used in the research were previously published [17].

### 2.4. Isolation of Protein Extracts and Western Blot Assay

Total protein extracts were isolated from cells treated with the compounds for 48 h by lysis with Laemmli buffer, followed by immediate protein denaturation by heating at 96 °C for 15 min. Protein concentration was assessed using the Pierce BCA Protein Assay Kit (Thermo Scientific, Waltham, MA, USA), and the absorbance was read using an Infinite M200 multi-plate reader (Tecan, Grödig, Austria). Protein content was further analyzed by Western blot.

Lysates were separated onto 7.5%, 10%, or 12% SDS-PAGE slab gels. Proteins were transferred to the nitrocellulose Immobilon P membrane (Sigma-Aldrich, St. Louis, MO, USA). After blocking for 2 h with 10% skimmed milk, proteins were probed with mouse anti-PFKM, mouse anti-PKM, mouse anti-LDHA, and rabbit anti-β-actin primary antibodies (Santa Cruz Biotechnology, Dallas, TX, USA). The horseradish peroxidase HRP-conjugated anti-mouse IgG or anti-rabbit IgG secondary antibodies (Bosterbio, Pleasanton, CA, USA) were used in the staining reaction. Bands were visualized using the chemiluminescent HRP Substrate Clarity ECL Kit (BioRad Laboratories, Hercules, CA, USA). The amount of immunoreactive products in each lane was determined using the ChemiDoc Imaging System (BioRad Laboratories, Hercules, CA, USA). Values were calculated as relative absorbance units (RQ) per mg of protein and expressed as a percentage of the control.

### 2.5. Bioinformatic Analysis of Gene Expression in Clinical Samples

Data available from the Cancer Genome Atlas (TCGA) were analyzed using the UALCAN tool (https://ualcan.path.uab.edu; accessed on 4 October 2023) [18,19] in order to evaluate the differences in the level of expression of *PFKM*, *PKM2*, and *LDHA* between healthy controls and HNSCC patients, and also between HPV-positive and HPV-negative HNSCC cases. Moreover, the changes in the promoter methylation level of these genes were assessed. Additionally, the association between glycolysis-related gene expression and HNSCC patient survival was determined using Kaplan–Meier plots generated in the UALCAN tool.

Furthermore, the UALCAN tool was used to analyze the changes in the level of expression of genes encoding the components of the Wnt pathway, which constitute molecular targets of the small-molecule inhibitors used in this study, and Wnt pathway-dependent transcription factors.

### 2.6. Bioinformatic Analysis of Protein Level in Clinical Samples

Protein level data available from the National Cancer Institute’s Clinical Proteomic Tumor Analysis Consortium (CPTAC) were analyzed using the UALCAN tool (https://ualcan.path.uab.edu; accessed on 4 October 2023) [18,19] to assess the differences in the level of expression of the enzymes related to the glycolytic pathway between healthy controls and HNSCC patients.

### 2.7. STRING Analysis

The STRING database (version 12.0) from the STRING CONSORTIUM 2023 (https://string-db.org; accessed on 5 October 2023) [20] was used to evaluate protein–protein interactions in order to show molecular inter-relationships between the Akt kinase and the Wnt/β-catenin pathway, as well as β-catenin and glycolysis.

### 2.8. Statistical Analysis

All data in this study were analyzed using GraphPad Instat, version 3.10 (GraphPad Software, San Diego, CA, USA) and are presented as mean ± SD. The differences between experimental groups were evaluated by a Student’s *t*-test (two groups) and one-way ANOVA test with the Tukey post hoc test (multiple groups). *p* < 0.05 was considered statistically significant.

## 3. Results

### 3.1. Higher Expression of PFKM and PKM2 Genes among HNSCC Patients Is a Negative Prognostic Marker of Survival Time

**Figure 1:**
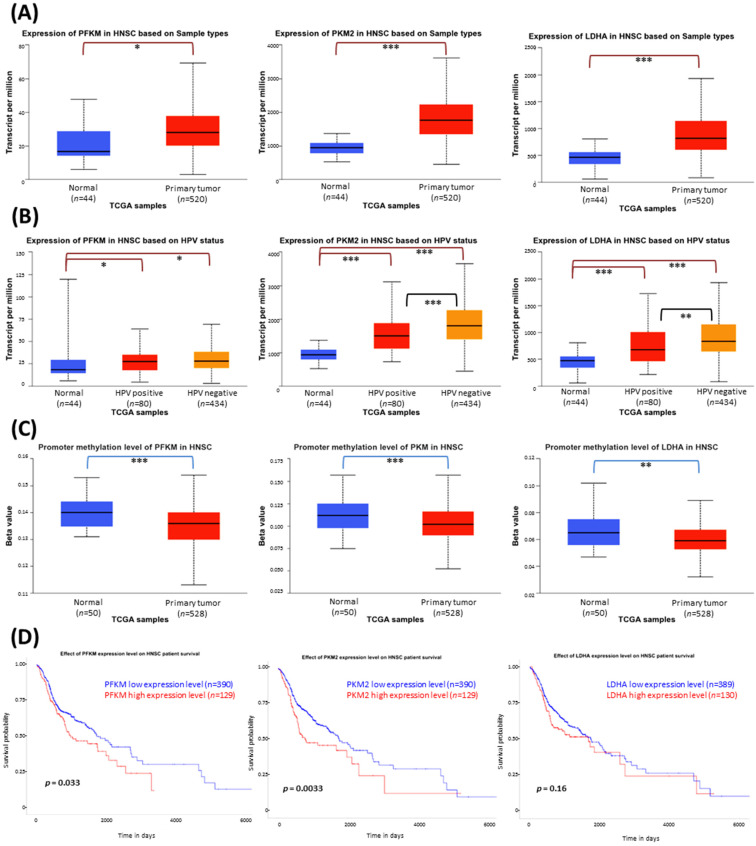
The results of the analysis of phosphofructokinase M (*PFKM*), pyruvate kinase M2 (*PKM2*), and lactate dehydrogenase A (*LDHA*) gene expression data available from the Cancer Genome Atlas using the UALCAN tool. (**A**) The differences in the level of gene expression between normal tissue and HNSCC samples. (**B**) The differences in the gene expression level depending on HPV status. (**C**) The differences in gene promoter methylation level between normal tissue and HNSCC samples. The Beta value indicates the level of DNA methylation ranging from 0 (unmethylated) to 1 (fully methylated). (**D**) Kaplan–Meier plots showing the association between high and low expression levels of glycolytic-related genes and survival time of HNSCC patients. The asterisk (*) denotes statistically significant changes, * *p* < 0.05, ** *p* < 0.01, *** *p* < 0.001.

Phosphofructokinase is a key enzyme controlling the early steps of glycolysis, while pyruvate kinase regulates the last step of pyruvate synthesis. Moreover, cancer cells using aerobic glycolysis require lactate dehydrogenase to produce energy and acidify the microenvironment. Based on the TCGA data, we checked the transcript expression level of isoenzymes which are important for cancer cells, i.e., PFKM, *PKM2*, and *LDHA*, among HNSCC patients (Figure 1A). For all three genes, the transcript level was significantly higher in HNSCC samples compared to normal tissues.

Head and neck cancers can develop independently of or due to HPV infection, which affects therapy outcomes. A higher level of the expression of *PKM2* and *LDHA* genes was shown in HPV-negative samples, which are more challenging to treat (Figure 1B). Thus, the Warburg effect may be an important factor in HPV-negative HNSCC progression.

One of the epigenetic mechanisms influencing gene expression is the DNA methylation of gene promoter regions. Although the basal methylation level of the analyzed genes was low (0.065–0.140), it was further significantly lowered in primary HNSCC tumor samples (Figure 1C).

Finally, Figure 1D presents Kaplan–Meier plots to compare the influence of high or low expression levels of particular glycolytic-related genes on the survival of HNSCC patients. The increased expression of *PFKM* (*p* = 0.033) and *PKM2* (*p* = 0.0033) negatively affects patient survival time. The impact of *LDHA* expression level was not statistically significant (*p* = 0.16), but an unfavorable trend can be observed.

### 3.2. The Protein Level of PKM and LDHA Is Increased in HNSCC Patient-Derived Cells

**Figure 2:**
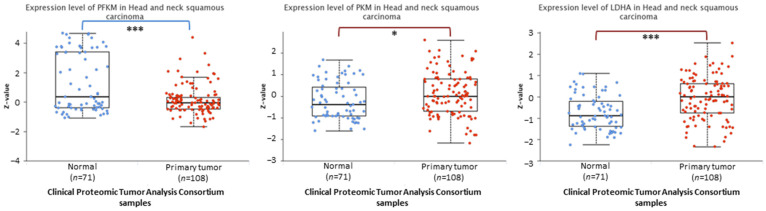
The results of the analysis of phosphofructokinase M (PFKM), pyruvate kinase M (PKM), and lactate dehydrogenase A (LDHA) protein expression data based on Clinical Proteomic Tumor Analysis Consortium (CPTAC) HNSCC samples using the UALCAN tool. Z-values represent standard deviations from the median across samples for the cancer type. Log2 Spectral count ratio values from CPTAC were first normalized within each sample profile and then normalized across samples. The asterisk (*) denotes statistically significant changes, * *p* < 0.05, *** *p* < 0.001.

In the next step, we analyzed whether the changes in transcript level translate into the protein expression level. Figure 2 presents data generated based on the National Cancer Institute’s Clinical Proteomic Tumor Analysis Consortium database. The expression of PKM and LDHA in HNSCC protein samples was indeed increased. In turn, the protein level of PFKM was significantly lower compared to normal samples, although its transcript level was upregulated (Figure 1A).

### 3.3. Wnt Signaling Pathway Elements Are Functionally Related to Akt Kinase and Glycolytic Enzymes

**Figure 3:**
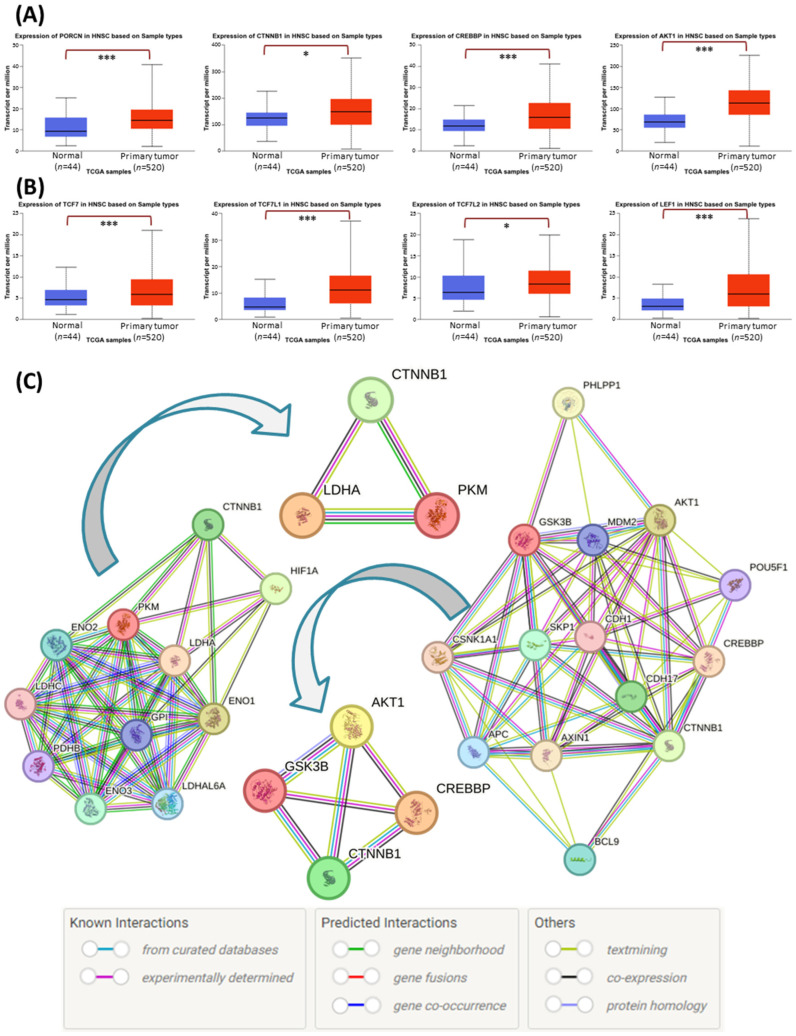
Analysis of Akt, Wnt signaling, and glycolytic pathway-related mRNA expression and functional protein–protein interactions. The results of the analysis based on data available from the Cancer Genome Atlas using the UALCAN tool showing the expression of: (**A**) genes being targets of small-molecule inhibitors used in the research, i.e., *PORCN*, *CTNNB1*, *CREBBP*, *AKT1*; (**B**) transcription factors responding to Wnt signaling activation, i.e., *TCF7*, *TCF7L1*, *TCFTL2*, *LEF1*. The asterisk (*) denotes statistically significant changes, * *p* < 0.05, *** *p* < 0.001. (**C**) The analyses of protein–protein interaction data available from the STRING database from the STRING CONSORTIUM 2023. The STRING graphs show the interaction of Wnt signaling elements with the Akt kinase and glycolysis proteins. Most important abbreviations: AKT1—Akt kinase 1, CREBBP—CREB binding protein, CTNNB1—β-catenin, GSK3B—glycogen synthase kinase 3β, LDHA—lactate dehydrogenase A, PKM—pyruvate kinase M, PORCN—porcupine.

Our previous research indicated the anticancer effects of Akt, Porcupine, β-catenin, and CREBBP inhibition in tongue squamous cell carcinoma cell lines [13]. Consistent with these results, the analysis of the TCGA data confirmed an increased level of the transcripts encoding these proteins (Figure 3A) in HNSCC clinical samples. Moreover, the higher mRNA levels of the four transcription factors (*TCF7*, *TCF7L1*, *TCF7L2*, *LEF1*) propagating the signals in the Wnt/β-catenin pathway (Figure 3B) also corroborate the pro-tumorigenic status of Wnt signaling in HNSCC.

In our previous studies, the inhibitors of the Wnt pathway (PRI-724 and IWP-O1) were able to modulate glucose utilization and lactate release in tongue cancer cells [13,16]. Moreover, the co-inhibition of Wnt signaling and the Akt kinase led to partly better effects than mono-treatment. Therefore, we wanted to evaluate the possible cross-talk between these signaling pathways (Wnt signaling and Akt kinase) and the glycolytic pathway by analyzing protein–protein interactions using the STRING database (Figure 3C). Many interactions were found for Akt kinase 1 and Wnt signaling elements: glycogen synthase kinase 3β (GSK3β), β-catenin (CTNNB1), and CREBBP. Thus, the simultaneous inhibition of the Akt kinase and Wnt pathway can potentially augment anticancer effects against HNSCC cells. In addition, CTNNB1 is directly related to PKM and LDHA, so it can potentially influence glycolytic flux. Thus, combining the Wnt pathway and Akt inhibitors could hypothetically enhance their suppressive effects against glycolytic enzyme expression. Therefore, in the next step, we performed quantitative real-time PCR analyses of *PFKM*, *PKM2*, and *LDHA* gene expression in tongue cancer cells to validate these hypotheses.

### 3.4. Wnt Inhibitors Reduce the Transcript Level of PFKM, PKM2, and LDHA

**Figure 4:**
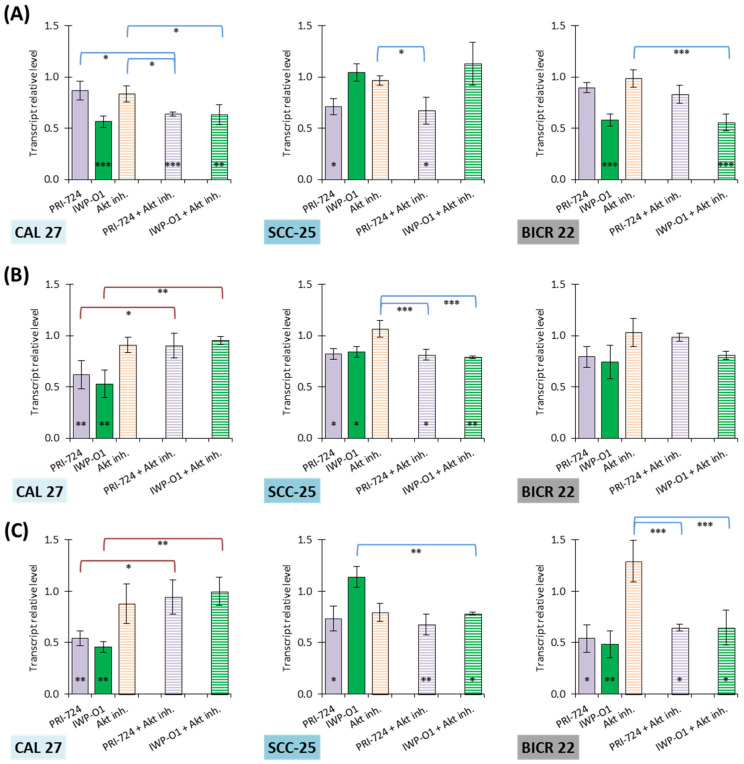
The effect of PRI-724, IWP-O1, and the Akt inhibitor on the relative transcript level of (**A**) phosphofructokinase M (*PFKM*), (**B**) pyruvate kinase M2 (*PKM2*), and (**C**) lactate dehydrogenase A (*LDHA*) in CAL 27, SCC-25, and BICR 22 cells. Mean values ± SD from three independent experiments with three replicates per qR-T PCR reaction are shown. The level of transcript in DMSO-treated cells was considered to be 1. The asterisk (*) inside the bar denotes statistically significant changes in comparison to the control, and above the bars denotes statistically significant changes in comparison to indicated experimental variants; * *p* < 0.05, ** *p* < 0.01, *** *p* < 0.001.

The inhibition of Wnt signaling lowered the mRNA expression of *PFKM*, particularly by IWP-O1 in CAL 27 and BICR 22 cells and PRI-724 in SCC-25 cells (Figure 4A). Individually used Akt inhibitors had no influence. The addition of Akt inhibitors to active Wnt inhibitors presented no additional effect on the *PFKM* transcript level, except a mixture of PRI-724 and Akt inhibitors in CAL 27 cells.

Both Wnt signaling inhibitors significantly reduced *PKM2* expression in CAL 27 and, to a lesser extent, SCC-25 cells (Figure 4B). Once again, the Akt inhibitor did not modulate the transcript level, and in combination, it eliminated the effects of Wnt pathway inhibitors (in CAL 27 cells) or had no influence (in SCC-25 cells). BICR 22 cells were resistant to all inhibitors.

Both Wnt signaling inhibitors importantly downregulated *LDHA* in CAL 27 and BICR 22 cells (Figure 4C). Moreover, PRI-724 slightly reduced the mRNA level in SCC-25 cells. The Akt inhibitor tended to change the expression of *LDHA*, but the results were insignificant. The combinations of PRI-724 or IWP-O1 with the Akt inhibitor showed no augmentation of effect or even worsened the effect (CAL 27 cells).

### 3.5. Wnt Signaling and Akt Kinase Inhibitors Strongly Downregulated LDHA Protein Expression

**Figure 5:**
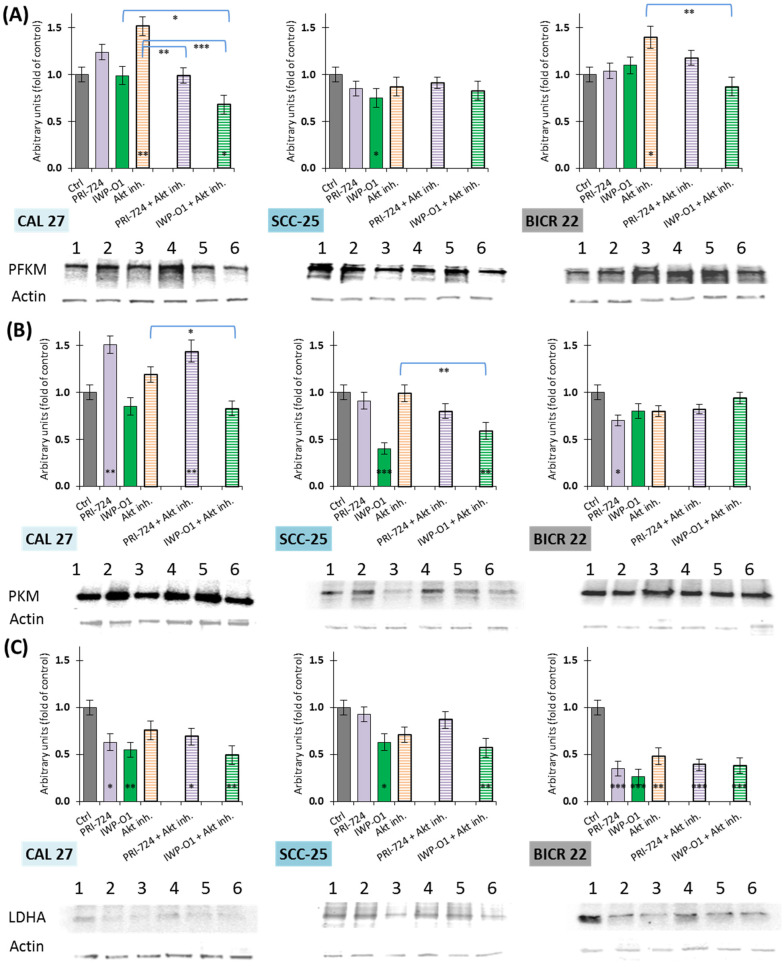
The effect of PRI-724, IWP-O1, and the Akt inhibitor on the relative protein level of (**A**) phosphofructokinase M (PFKM), (**B**) pyruvate kinase M2 (PKM2), and (**C**) lactate dehydrogenase A (LDHA) in CAL 27, SCC-25, and BICR 22 cells. Mean values ± SD from two independent experiments are shown. The level of protein in DMSO-treated cells was considered to be 1. The asterisk (*) inside the bar denotes statistically significant changes in comparison to control, and above the bars denotes statistically significant changes in comparison to indicated experimental variants; * *p* < 0.05, ** *p* < 0.01, *** *p* < 0.001. Exemplary immunoblots are presented below the plots. 1—Ctrl, 2—PRI-724, 3—IWP-O1, 4—Akt inhibitor, 5—PRI-724 + Akt inhibitor, 6—IWP-O1 + Akt inhibitor.

In the next step, we analyzed whether the changes in transcript expression levels elicited by the studied chemicals were followed by changes in the level of respective proteins (Figure 5). For individual compounds, the protein level of PFKM was significantly reduced only after the exposure of SCC-25 cells to the IWP-O1 inhibitor. On the contrary, the Akt inhibitor increased the expression of this enzyme in CAL 27 and BICR 22 cells. Interestingly, in CAL 27 cells, the combination of IWP-O1 and the Akt inhibitor reduced the PFKM protein level by ~32%.

Porcupine inhibitor IWP-O1 significantly reduced the expression of PKM in SCC-25 cells. In parallel, PRI-724 increased the PKM protein level in CAL 27 cells, was neutral in SCC-25 cells, and lowered its expression in BICR 22 cells. Co-treatment of cells with Wnt inhibitors and the Akt inhibitor did not show beneficial effects, while in SCC-25 cells, the expression level of PKM for IWP-O1 and the Akt inhibitor mixture was higher compared to individual IWP-O1 use. CAL 27 cells exposed to PRI-724 and the Akt inhibitor presented comparably high protein levels compared to PRI-724 alone.

Lactate dehydrogenase A was most susceptible to downregulation. In BICR 22 cells, each experimental variant significantly decreased the LDHA protein level. Furthermore, in CAL 27 cells, only the Akt inhibitor had an insignificant influence on LDHA expression. In turn, SCC-25 cells were affected by IWP-O1 and its combination with the Akt inhibitor. We did not detect notably better results of combinatorial treatments in comparison to exposure to single inhibitors in any of the cell lines.

## 4. Discussion

Changes in energy metabolism, known as the Warburg effect, are part of the characteristics of HNSCC cells. Indeed, glucose was identified as the dominant energy source for this type of tumor [21]. Thus, balancing glycolytic activity comprises a valid molecular target for HNSCC therapy. Previously, we described the anti-glycolytic effects of 2-deoxyglucose and Hif-1α and c-Myc inhibitors in hypopharyngeal FaDu cells [17]. In other studies, we detected a significant influence of PRI-724 and IWP-O1 Wnt signaling inhibitors on glucose utilization by tongue cancer cells [13,16]. Therefore, in this study, we further explored the association between the inhibition of Wnt signaling and/or the Akt kinase and the expression of crucial glycolysis-related genes in tongue cancer cells, in order to better recognize the interconnections between these processes.

Phosphofructokinase M, pyruvate kinase M2, and lactate dehydrogenase A were selected as pivotal isoenzymes in the glucose flux during aerobic glycolysis. Based on the TCGA database, we showed that all of them are overexpressed at the transcript level among HNSCC patients. Although the promoter methylation percentage was generally low, its value was slightly decreased in HNSCC tissues, which can favor gene expression. In addition, *PKM2* and *LDHA* genes present higher transcript copy numbers per million in HPV-negative cases, suggesting a greater metabolic disturbance in this group of difficult-to-treat tumors. Moreover, these molecular events have clinical importance, since high *PFKM* and *PKM2* mRNA expression correlates with worse prognosis, i.e., shorter survival of patients.

Activated Wnt signaling has been implicated in the enhancement of glycolytic activity in cancer cells, e.g., in nasopharyngeal carcinoma via the upregulation of pyruvate dehydrogenase kinase 1 [22] or by increasing glucose uptake in hepatocellular carcinoma [23]. The influence of Wnt1-inducible signaling protein 1 (WISP1) on glucose uptake, glycolytic flux, and lactate production was additionally observed in laryngeal squamous cell carcinoma [24]. Recently, Huang et al. (2023) pointed to DEP domain containing 1 (DEPDC1) as the glycolysis-related biomarker associated with oral squamous cell carcinoma progression. Such an effect is mediated by the activation of the Wnt/β-catenin signaling pathway [25].

Based on TCGA database analysis, we showed that *PORCN*, *CTNNB1*, and *CREBBP* genes are overexpressed in HNSCC, similarly to *AKT1* and four transcription factors acting downstream of the Wnt/β-catenin signaling pathway, which further corroborates the aberrant activation of Wnt signaling. Next, we performed the bioinformatic analysis of protein–protein interactions between glycolytic enzymes and Wnt pathway proteins. The STRING plots showed a three-sided relation between β-catenin, PKM, and LDHA, and the interaction between the Akt kinase and Wnt signaling elements, i.e., GSK-3β, β-catenin, and CREBBP. Indeed, the Akt/Wnt/β-catenin axis seems vital to the development of HNSCC [26,27,28]. Such data theoretically corroborate the possibility that Wnt inhibitors could decrease glycolytic activity in HNSCC cells, and that the combination of the Wnt pathway and Akt kinase inhibitors could improve the therapeutic effects of individual inhibitors.

We used two Wnt pathway inhibitors with distinct mechanisms of action to experimentally validate the influence of Wnt inhibition on the expression of glycolytic genes. PRI-724 blocks the interaction between β-catenin and CREBBP in the nucleus, while IWP-O1 targets porcupine responsible for the maturation of Wnt ligands. These small-molecule inhibitors of Wnt signaling were able to downregulate *PFKM*, *PKM2*, and *LDHA* gene expression, which is in line with the interaction between the Wnt pathway (β-catenin) and glycolytic genes. Conversely, individually used Akt inhibitors had no meaningful influence on transcript levels. Almost no additional reduction was observed in combinations with a Wnt signaling inhibitor, except for a mixture with PRI-724 in CAL 27 cells. Therefore, the assumption of the enhancement of anti-glycolytic effects by simultaneous targeting of Akt and Wnt signaling was not confirmed by our experimental results. We also performed protein level analysis of PFKM, PKM2, and LDHA. IWP-O1 showed a higher ability to decrease the levels of proteins of interest. Furthermore, in CAL 27 cells, the combination with Akt inhibitor enhanced its activity and reversed the direction of Akt inhibitor influence. However, the protein expression analysis did not confirm the activity of the PRI-724 inhibitor in most cases, and even the direction of effect was the opposite. On the other hand, the PRI-724 inhibitor of the β-catenin–CREBBP interaction was the most effective in reducing glucose uptake and lactate release in the same tongue cancer cell lines [13]. We suppose that additional molecular mechanisms, like posttranslational modifications, can orchestrate the final effect on glycolysis. For instance, the phosphorylation of Tyrosine 105 in PKM2 was described as a frequent molecular event in HNSCC cells correlated with high glycolytic activity [29].

Aberrant Wnt signaling modulates glycolysis, favoring lactate production, and other metabolic processes, including glutaminolysis and lipogenesis. By altering metabolism, which leads to extracellular fluid acidification, the Wnt pathway can actively regulate the tumor microenvironment, negatively modify the immune response, and promote tumor development [30]. Interestingly, β-catenin induces the expression of activating transcription factor 3 (ATF3) and inhibits the transcription of C-C motif chemokine ligand 4 (CCL4), which results in the impaired infiltration and activation of ATF3-related CD103+ dendritic cells, reduced CD8+ effector T cell priming and infiltration, and finally, a lack of proper responses to immune checkpoint blockade [31]. Argentiero et al. (2019) compared in silico RNA-seq data of 64 lymph node-positive and 79 lymph node-negative pancreatic ductal adenocarcinoma (PDAC) patient-derived samples [32]. Notably, the altered expression of a cluster covering the Wnt signaling pathway-related genes was associated with pleiotropic effects on immune response, epithelial modeling, and cytokine regulation in lymph node-positive PDAC cases. Lymph node-positive PDAC patients showed an increased number of activated dendritic cells and M2 macrophages with a concomitant decrease in effector T cells. Further experiments using PDAC lymph node-positive PANC-1 cells, PDAC lymph node-negative MIAPaCa-2 cells, and XAV-939 Wnt signaling inhibitor demonstrated, e.g., increased cytotoxic and antiproliferative effects of the Wnt inhibitor when PDAC cells were co-cultured with peripheral blood mononuclear cells. In addition, the inhibitor of Wnt signaling restored the expression of the macrophage stimulating 1 (MST-1) protein related to cancer suppression via the production of reactive oxygen species and inhibited angiopoietin-like 4 (ANGPTL4) expression related to angiogenesis, tumor migration, and regulation of immune homeostasis [32]. More information about Wnt pathway connections with immune cells, immunotherapy, and tumor microenvironments can be found, e.g., in [33,34,35,36,37]. The influence of Wnt signaling on metabolic and immune changes is an excellent target for future research on anti-cancer therapy.

The experiments described in this article confirmed that Wnt signaling inhibitors have a significant role in the attenuation of glycolysis in tongue squamous cancer cells. The inhibitory influence on PFKM, PKM2, and LDHA expression generally supports the metabolic effects observed in our previous work, in which we have shown the changes in glucose consumption, lactate release, and cellular ATP level [13]. Conversely, the inhibition of the Akt kinase may have a lower effect on glucose metabolism in tongue tumor cells than initially thought. This study was limited to assessing the expression of three crucial glycolytic genes. As the conclusions derived from our in vitro model are limited, future studies will thus need to include cell lines from different HNSCC localizations, patient-derived primary cells, and three-dimensional organoids in co-culture with other cell types present in the tumor microenvironment. Moreover, we have not evaluated the long-term effects of the studied inhibitors, or the potential resistance mechanisms that can affect the treatment outcome, and these factors will need to be addressed in the future to optimize the anti-cancer effects of the chemicals. Nevertheless, the observed correlation between Wnt signaling and energy metabolism suggests a more comprehensive effect of drugs targeting this pathway on the hallmarks of HNSCC cancer cells.

## Data Availability

Data are contained within the article.
